# Risk of postoperative hypoxemia in ambulatory orthopedic surgery patients with diagnosis of obstructive sleep apnea: a retrospective observational study

**DOI:** 10.1186/1754-9493-4-9

**Published:** 2010-06-21

**Authors:** Spencer S Liu, Mary F Chisholm, Raymond S John, Justin Ngeow, Yan Ma, Stavros G Memtsoudis

**Affiliations:** 1 Department of *Anesthesiology, Hospital for Special Surgery, Weill College of Medicine of Cornell University, 535 East 70th Street, New York, NY 10021, USA; 2 Department of Public Health, Weill Medical College of Cornell University, New York, USA

## Abstract

**Background:**

It is unclear when it is safe to discharge patients with a diagnosis of Obstructive Sleep Apnea (OSA) after ambulatory surgical procedures due to concern for postoperative respiratory compromise and hypoxemia. Our OSA patients undergoing ambulatory-type orthopedic procedures are monitored overnight in the PACU, thus we reviewed patient records to determine incidence of complications.

**Methods:**

Two hundred and six charts of patients with preoperative diagnosis of OSA based on ICD-9 codes were reviewed for outcomes including episodes of hypoxemia. Univariate analysis followed by logistic regression and propensity analysis was performed to determine independent risk factors for hypoxemia and association with adverse outcomes.

**Results:**

The majority of patients had regional anesthesia (95%). Thirty four percent of patients had hypoxemia in the PACU. Initial risk factors for hypoxemia identified by univariate analysis were BMI ≥ 35, increased age, history of COPD, upper extremity procedure, and use of peripheral nerve block. Independent risk factors identified by logistic regression were history of COPD (OR 3.64 with 95% CI 1.03-12.88) and upper extremity procedure (2.53, 1.36-4.68). After adjustment with propensity scores, adverse events were rare, and unplanned hospital admission after PACU stay was not increased with hypoxemia (11% vs 16%)

**Conclusions:**

Episodes of postoperative hypoxemia in OSA patients undergoing ambulatory surgery with regional anesthesia are not associated with increased adverse outcomes or unplanned hospital admission.

## Background

Ambulatory anesthesia is increasing in worldwide popularity, and approximately 60% of procedures in the United States in 2007 were performed on an ambulatory basis [1]. However, it remains unclear whether it is safe to immediately discharge patients with a diagnosis of obstructive sleep apnea (OSA) immediately home after ambulatory procedures. OSA is associated with increased morbidity due to hypoxemia, and estimates for prevalence of OSA range from 10-64% [2]. Postoperative apnea may be more severe due to perioperative disturbances in sleep architecture [3] and respiratory depressant effects of postoperative analgesics [4]. Current clinical guidelines for OSA are based only on expert opinion, [2,5,6] and incidence of respiratory compromise after ambulatory procedures in OSA patients is unknown [7]. Hospital for Special Surgery is an orthopedic surgical hospital and has had a policy since December, 2005 that patients with a diagnosis of OSA spend the night in our post anesthesia care unit (PACU) for continuous monitoring including pulse oximetry after ambulatory surgical procedures. Thus, we retrospectively reviewed charts of patients with a preoperative diagnosis of OSA to determine if there was an association with hypoxemia and adverse outcomes including unplanned subsequent hospital admission.

## Methods

After obtaining Institutional Review Board (IRB) approval, 206 patients with a preoperative diagnosis of OSA undergoing ambulatory surgery from December, 2005 to March, 2009 were identified by using ICD-9 codes 327.23 and 780.57 for OSA. Their charts were reviewed and retrospective data abstracted. Since the research did not present more than minimal risk of harm to the subjects or their privacy, the IRB granted waivers for informed consent and Health Insurance Portability and Accountability Act (HIPAA) authorization. All patients spent at least the first postoperative night in the PACU with initially 2 liters/min oxygen delivery via nasal canula. All patients were continually monitored with pulse oximetry and other standard non-invasive monitors. Collected data included patient characteristics, type of procedure, type of anesthetic, intraoperative data, postoperative course and complications during hospitalization, and type of postoperative analgesia. Hypoxemia was defined as a Spo2 < 95% on pulse oximetry.

### Statistical analysis

Episode of hypoxemia was the primary outcome. Descriptive analysis was initially performed to determine if any differences in perioperative characteristics (Table 1) were apparent between groups that had at least one episode of hypoxemia (mild or severe) versus those who did not. Perioperative characteristics that appeared to differ between groups were then compared with univariate analysis (t test or chi square test) to screen for potential risk factors for hypoxemia. Potential risk factors that had a p < 0.05 on univariate analysis were then used for multivariate logistic regression to determine independent risk factors for hypoxemia (mild or severe). Logistic regression was performed with both discrete and continuous predictors and was adjusted for type of procedure. A combination of forwards and backwards stepwise methods was used for logistic model selection.

**Table 1:** Selected perioperative characteristics

Characteristic	No hypoxemia (n = 135)	Hypoxemia (n = 71)
Age *(yrs, mean/SD)	55 ± 11	58 ± 12

Gender (M/F, %)	79%/21%	77%/23%

BMI (mean/SD)*	32 ± 6	35 ± 7

ASA physical status (1/2/3/4, %)	1/61/37/1	1/47/52/0

**Past Medical History**		

CPAP at home (%)	53%	54%

COPD (%)*	3%	11%

Procedure*		

Knee arthroscopy (%)	25%	18%

Knee ACL Repair (%)	3%	3%

Hand/Forearm (%)	12%	20%

Shoulder arthroscopy (%)	33%	47%

Foot/Ankle (%)	12%	7%

Other upper extremity (%)	4%	4%

Other lower extremity (%)	11%	1%

Anesthesia*		

General only (%)	6%	4%

General + regional (%)	3%	3%

Regional only	91%	93%

Spinal/Epidural (%)	46%	28%

Interscalene block (%)	25%	35%

Other nerve block (%)	21%	31%

**Postoperative Analgesia**		

Oral Opioids (%)	85%	92%

NSAIDs (%)	36%	39%

IV Opioids (%)	24%	32%

* = Identified as potential risk factor by univariate analysis

Upper extremity vs lower extremity. Regional vs General

Unadjusted association between hypoxemia with adverse outcomes was tested with either Chi square or T test. Adjusted association between hypoxemia with adverse outcomes was examined with propensity analysis to reduce bias between groups with and without hypoxemia. Propensity scores for each patient were created based on the above identified risk factors for hypoxemia [8]. A propensity score is the probability of assignment to a particular condition based on a set of known covariates, and propensity analysis performs statistical adjustment with propensity scores to reduce selection bias by equating patients based on these covariates. Thus, propensity analysis was performed by logistic regression with propensity scores and hypoxemia as independent variables against any adverse outcome that had unadjusted statistical differences between groups with and without hypoxemia [9,10]. P < 0.05 was considered as significant for all analyses.

## Results

Table 1 displays selected perioperative characteristics. As expected in an exclusively OSA population, [6] our patients were predominantly male (78%), older (mean age 56), overweight (101 kg mean), and 54% used CPAP at home. The majority (95%) of patients underwent regional anesthesia. In the PACU, thirty four percent of patients had at least one episode of hypoxemia. Potential risk factors for hypoxemia identified by univariate analysis were BMI ≥ 35, increased age, history of chronic obstructive pulmonary disease (COPD), upper extremity procedure, and use of peripheral nerve block. From these, independent risk factors identified by logistic regression were history of COPD (OR 3.64 with 95% CI 1.03-12.88) and upper extremity procedure (2.53, 1.36-4.68). The Hosmer and Lemeshow test was performed and indicated a good fit of the data for the regression model (p = 0.87). Incidences of hypoxemia (22-78%) varied with combinations of risk factors (Table 2).

**Table 2:** Incidence and risk of hypoxemia for individual and combinations of risk factors

Number of risk factors	Risk factors combinations	Incidence of hypoxemia (% and number of pts)	Odds
0	None	22% (20)	0.35

1	COPD	33% (1)	0.5
	
	Upper extremity procedure	41% (43)	0.71

2	COPD + Upper extremity procedure	78% (7)	2.59

Odds were calculated from logistic regression. Individual odds ratios can be calculated for any pair. For example the odds ratio for both risk factors versus just COPD is (2.59/0.5 = 5.18)

None of our patients suffered from major complications. Incidences of postoperative hypertension, need for continuous positive airway pressure (CPAP), and need for hospital admission after required PACU stay were not associated with hypoxemia (Table 3). Only a need for increased oxygen flow was significantly associated with hypoxemia which remained significant after propensity analysis.

**Table 3:** Adverse outcomes

Outcome	No hypoxemia (N = 135)	Hypoxemia (N = 71)
CPAP at home + subsequent routine PACU use (%)	33	41

Emergent CPAP (%)	0	0

Increase in oxygen delivery (%)	16	37*

Incidence of postoperative hypertension (%)	33	41

Need treatment for hypertension (%)	4	4

Need for postoperative hospital admission from PACU (%)	16	11

*Reasons for hospital admission*		

Physical Therapy (%)	45	13

Intravenous antibiotics (%)	9	13

Pain Control (%)	14	13

Infectious Complication (%)	18	38

Radiation therapy (%)	0	13

Other (%)**	14	13

* = P < 0.05. ** No patients admitted for persistent hypoxemia

## Discussion

Episodes of postoperative hypoxemia in OSA patients undergoing ambulatory surgery were not associated with increased adverse outcomes or unplanned hospital admission. The only previous study of OSA patients undergoing ambulatory surgery was a retrospective case series that matched 234 patients with OSA (diagnosed with polysomnography) against an equal number of normal patients, [11] but did not perform overnight respiratory monitoring as in our study. No patients suffered from a serious adverse event, and similar rates of unplanned hospital admission (24% vs. 19%) were reported for the two groups. Our rates of adverse outcomes and unplanned hospital admission were similar to this previous study,[11] which suggests external validity for our data. As neither a diagnosis of OSA nor suffering from an episode of hypoxemia is associated with increased risk of adverse outcomes, it may be reasonable to discharge patients with a diagnosis of OSA home after regional anesthesia for ambulatory orthopedic surgery.

Our data provide support for current consensus based clinical guidelines for OSA patients undergoing ambulatory anesthesia [5]. Peripheral and minor procedures, use of regional anesthesia, and limited requirement for strong postoperative opioids are all considered to decrease risk per these guidelines [5]. Our procedure types (ambulatory orthopedic), predominant use of regional anesthesia (95% of procedures), and modest requirement for postoperative intravenous opioids (0-36% of patients) likely decreased the inherent risk for complications in these patients to the point that hypoxemia from OSA was no longer a significant negative event.

Our surgical population underwent exclusively elective ambulatory orthopedic procedures such as knee and shoulder arthroscopy which are frequently performed and growing in volume. The latest data set (2009) from the National Survey of Ambulatory Surgery from the National Center for Health Statistics reported that greater than 39 million ambulatory surgical procedures are performed annually in the United States and that musculoskeletal procedures such as orthopedics are the 2^nd ^most commonly performed type of procedures [12]. Ambulatory surgery is increasingly popular worldwide, thus our findings should have widespread clinical applicability.

There are several limitations to our study. The data collection was retrospective and has typical limitations in that data may have been missed or miscoded. The diagnosis of OSA was based on ICD9 codes, thus severity of OSA could not be ascertained. Few patients underwent general anesthesia, thus results can not be extrapolated to such patients.

## Conclusions

Episodes of postoperative hypoxemia in OSA patients undergoing ambulatory surgery with regional anesthesia were not associated with increased adverse outcomes or unplanned hospital admission. Our OSA patients had similar rates of unplanned hospital admission as previously reported in normal patients. Thus, this study provides original data that support current clinical OSA guidelines recommending that such patients do not require overnight monitored observation.

## Competing interests

The authors declare that they have no competing interests.

## Authors' contributions

SSL conceived the original design of the study, interpreted data, and drafted the manuscript. RSJ and JN carried out and coordinated the study design, acquired data, and assisted with the revision of the final manuscript. MFC and SGM participated in the conception and design of the study and helped draft and revise the manuscript. YM performed the statistical analysis and helped draft and revise the manuscript. All authors read and approved the final manuscript.

## References

[B1] Chanthong P Abrishami A Wong J Herrera F Chung F Systematic review of questionnaires measuring patient satisfaction in ambulatory anesthesia Anesthesiology 2009 110 1061 1067 10.1097/ALN.0b013e31819db079 19352161

[B2] Chung SA Yuan H Chung F A systemic review of obstructive sleep apnea and its implications for anesthesiologists Anesth Analg 2008 107 1543 1563 10.1213/ane.0b013e318187c83a 18931212

[B3] Gogenur I Wildschiotz G Rosenberg J Circadian distribution of sleep phases after major abdominal surgery Br J Anaesth 2008 100 45 49 10.1093/bja/aem340 18037670

[B4] Bernards CM Knowlton SL Schmidt DF DePaso WJ Lee MK McDonald SB Bains OS Respiratory and sleep effects of remifentanil in volunteers with moderate obstructive sleep apnea Anesthesiology 2009 110 41 49 10.1097/ALN.0b013e318190b501 19104169

[B5] Gross JB Bachenberg KL Benumof JL Caplan RA Connis RT Coté CJ Nickinovich DG Prachand V Ward DS Weaver EM Ydens L Yu S Practice guidelines for the perioperative management of patients with obstructive sleep apnea: a report by the American Society of Anesthesiologists Task Force on Perioperative Management of patients with obstructive sleep apnea Anesthesiology 2006 104 1081 1093 10.1097/00000542-200605000-00026 16645462

[B6] Isono S Obstructive sleep apnea of obese adults: pathophysiology and perioperative airway management Anesthesiology 2009 110 908 921 10.1097/ALN.0b013e31819c74be 19293689

[B7] Bryson GL Chung F Finegan BA Friedman Z Miller DR van Vlymen J Cox RG Crowe MJ Fuller J Henderson C Patient selection in ambulatory anesthesia - an evidence-based review: part I Can J Anaesth 2004 51 768 781 10.1007/BF03018449 15470165

[B8] Rosenbaum PR Rubin DB The central role of the propensity score in observational studies for causal effects Biometrika 1983 70 41 55 10.1093/biomet/70.1.41

[B9] Nuttall GA Houle TT Liars, damn liars, and propensity scores Anesthesiology 2008 108 3 4 10.1097/01.anes.0000296718.35703.20 18156873

[B10] D'Agostino RB Jr Propensity score methods for bias reduction in the comparison of a treatment to a non-randomized control group Stat Med 1998 17 2265 2281 10.1002/(SICI)1097-0258(19981015)17:19<2265::AID-SIM918>3.0.CO;2-B 9802183

[B11] Sabers C Plevak DJ Schroeder DR Warner DO The diagnosis of obstructive sleep apnea as a risk factor for unanticipated admissions in outpatient surgery Anesth Analg 2003 96 1328 1335 table of contents 10.1213/01.ANE.0000061585.09157.66 12707128

[B12] Ambulatory Surgery in the United States, 2006 http://www.cdc.gov/nchs/data/nhsr/nhsr011.pdf

